# Effects of mineral complex material treatment on 2,4- dinitrochlorobenzene-induced atopic dermatitis like-skin lesions in mice model

**DOI:** 10.1186/s12906-021-03259-5

**Published:** 2021-03-03

**Authors:** Johny Bajgai, Jing Xingyu, Ailyn Fadriquela, Rahima Begum, Dong Heui Kim, Cheol-Su Kim, Soo-Ki Kim, Kyu-Jae Lee

**Affiliations:** 1 Department of Environmental Medical Biology, Wonju College of Medicine, Yonsei University, Wonju, Gangwon-do 26426 Republic of Korea; 2 Department of Global Medical Science, Graduate School, Yonsei University, Wonju, Gangwon-do 26426 Republic of Korea; 3 Department of Laboratory Medicine, Wonju College of Medicine, Yonsei University, Wonju, Gangwon-do 26426 Republic of Korea; 4 Department of Microbiology, Wonju College of Medicine, Yonsei University, Wonju, Gangwon-do 26426 Republic of Korea; 5 Institute for Poverty Alleviation and International Development, Yonsei University, Wonju Campus, Wonju, Gangwon-do 26493 Republic of Korea

**Keywords:** Atopic dermatitis, Inflammatory disease, Mineral complex material patch, Dinitrochlorobenzene, Immune redox

## Abstract

**Background:**

Atopic dermatitis (AD) is a chronic allergic inflammatory skin disease characterized by complex pathogenesis including skin barrier dysfunction, immune-redox disturbances, and pruritus. Prolonged topical treatment with medications such as corticosteroids, calcineurin inhibitors, and T-cell inhibitors may have some potential side-effects. To this end, many researchers have explored numerous alternative therapies using natural products and mineral compounds with antioxidant or immunomodulatory effects to minimize toxicity and adverse-effects. In the current study, we investigated the effects of mineral complex material (MCM) treatment on 2, 4-dinitrochlorobenzene (DNCB)-induced AD-like skin lesions in SKH-1 hairless mice.

**Methods:**

Animals were divided into four groups; normal control (NC), negative control treated with DNCB only (DNCB only), positive control treated with DNCB and tacrolimus ointment (PC) and experimental group treated with DNCB and MCM patch (MCM). Skin inflammation and lesion severity were investigated through analyses of skin parameters (barrier score and strength, moisture and trans-epidermal water loss level), histopathology, immunoglobulin E, and cytokines. In addition, reactive oxygen species (ROS), nitric oxide (NO), glutathione peroxidase (GPx), and catalase (CAT) levels were measured in both serum and skin lysate.

**Results:**

Our results demonstrates that MCM patch improved the progression of AD-like skin lesions by significantly increasing skin barrier strength and decreasing trans-epidermal water loss. Additionally, dermal administration of MCM patch significantly reduced epidermal thickness, ROS, and NO levels in skin lysate. Furthermore, we found that MCM suppressed the levels of AD-involved (Th_1_ and Th_2_) cytokines such as IL-2, IFN-γ, and IL-4 in blood. In addition, the levels of other Th_1,_ and Th_2_ and inflammatory cytokines such as IL-1β, TNF-α, IL-6, IL-12(p70) and IL-10 were found lowest in the MCM group than in the DNCB only and PC groups. Moreover, we found total serum IgE level significantly increased after DNCB treatment, but decreased in the PC and MCM groups.

**Conclusion:**

Taken together, our findings suggest that MCM application may have beneficial effects either systemic or regional on DNCB-induced AD lesional skin via regulation of the skin barrier function and immune-redox response.

**Supplementary Information:**

The online version contains supplementary material available at 10.1186/s12906-021-03259-5.

## Background

Atopic dermatitis (AD) is a chronic inflammatory skin disease characterized by itchy skin, erythematous lesions, and increased trans-epidermal water loss (TEWL); this disease is also known as atopic eczema [1, 2]. Approximately 10 to 20% of infants and young children and 1 to 3% of adults all over the world are affected by AD [3]. The detailed mechanism of AD is not entirely clear, but studies have indicated complex pathophysiology involving disruption of the skin barrier function, dysfunctional immunological system, immunoglobulin E (IgE)-mediated hypersensitivity, as well as psychological, genetic, and environmental factors [3, 4]. Besides that, the role of oxidants such as reactive oxygen species (ROS) and reactive nitrogen species (RNS) are found to be important contributing factors in the pathogenesis of AD as a result of oxidative stress. Excessive productions of these oxidants are controlled by various enzymatic antioxidants such as glutathione peroxidase (GPx) and catalase (CAT) activities [5]. Increasing evidence has shown that AD skin inflammation is regulated by a variety of inflammatory cell mediators such as T lymphocytes, dendritic cells expressing IgE, and cytokines expressed by T helper cell type 1 (Th1) and Th2 cells [1, 6]. A variety of cytokines such as interleukin (IL)-2, IL-12, interferon γ (IFN γ) and tumor necrosis factor α (TNF α) are secrete by Th1 cells to trigger macrophages, while cytokines such as IL-4, IL-5, IL-6, IL-10 and IL-13 are secrete by Th2 cells to increase IgE production and induce mast cell and eosinophil differentiation [7, 8]. During contact to pathological stimuli, IL-1β is produced by activated leukocytes, leading to induction and enhancement of inflammatory responses in inflammatory diseases such as AD [9]. Under normal conditions, interaction between the Th1 and Th2 cells balances and maintains the immune activities. However, during onset of AD, Th2 cells secrete an excessive number of inflammatory cytokines and trigger to release an increase serum IgE, further promoting the inflammatory activities [10–12]. To treat the disease, immune- modulating therapies have been developed based on the etiology of AD, such as calcineurin inhibitors, corticosteroids, T-cell inhibitors, and emollients; however, prolonged usage of these agents often produce adverse effects [13, 14]. Therefore, there is a great need to develop for new safer, efficient therapies to control the symptoms of AD.

Over the past few centuries, researchers have had great interest in exploring the precise roles of natural compounds such as water, rocks, minerals, and trace elements in animal and human health as well as various diseases; however, there have been only few multidisciplinary studies investigated on the relationship between these materials on animal and human health [15, 16]. In previous reports, many ore minerals have been investigated for its efficacy against skin inflammatory diseases, stomatitis, periodontal diseases, and many other diseases because of their high absorptive capacity, chemical inertness, and low or no toxic properties [15–19]. In traditional Korean medicine minerals from ores have been used for decades to treat diseases such as dysentery, dry breast, poor micturition and chronic indigestion; in fact, as many as 92 medicinal ores are recorded Dongeuibogam, a medicinal book written by Dr. Heo Jun [20]. In addition, our previous in vivo study has shown that application of natural ore minerals significantly improve skin and immune-redox parameters against UVB-induced skin damage [21]. Furthermore, cumulative studies have shown that mineral therapy might have therapeutic effects on skin diseases in humans and inflammatory disease in mouse models [22–26]. Another study reported that mineral therapy not only significantly reduces epidermal hyperplasia and inflammatory cell infiltration but also modulates immune cell activities [27].

Tourmaline is a ring- shaped borosilicate complex ore mineral with crystalline compound and molecular formula of Na (Mg, Fe, Mn, Li, Al) _3_Al _6_(BO_3_)_3_Si_6_O_18_ (O, OH, F)_4_; it contains trace elements such as V, Cr, Zr, Mn, Ti, Sr and Ga. This complex mineral compound has characteristics of far-infrared radiation emission, spontaneous polarizability, and protective effects against electromagnetic wave, and anion –releasing effect; it is widely used in healthcare facilities [28–30]. Numerous studies have shown that emission of infrared and negative ions from tourmaline has positive effects against several inflammatory diseases and dermatological diseases including psoriasis and AD [30–34]. Moreover, Zou and colleagues reported that tourmaline-containing film showed beneficial effects in the healing of burn wound in vitro and in vivo [35]. However, despite of this evidence in the field of mineralogy, the mechanism underlying the therapeutic effects of these complex ore minerals on AD has not been investigated in detail. In the present study, we explored the therapeutic effects of mineral complex material (MCM) patch made of natural ore powders containing mainly tourmaline and other complex minerals (Table 1), on AD-like lesions in SKH-1 hairless mice induced by 2, 4-dinitrochlorobenzene (DNCB). We hypothesized that MCM exerts therapeutic effects via regulation of the skin barrier function and immune-redox response. To further verify our hypothesis, we investigated physiological and morphological skin parameters as well as immune-redox and allergic profiles after MCM patch-treatment in mice with DNCB-induction.

**Table 1:** Composition analysis of natural ore powder as the material of MCM patch

Material	Component Name	Chemical Formula	CAS Number	Content [wt%]
Natural ore material (powder)	Silicon dioxide	SiO_2_	7631-86-9	<1 ~ 30%
	Aluminum oxide	Al_2_O_3_	1344-28-1	<1 ~ 30%
	Magnesium oxide	MgO	1309-48-4	<1 ~ 20%
	Titanium dioxide	TiO_2_	13,463–67-7	<1 ~ 20%
	Potassium oxide	K_2_O	12,136–45-7	<1 ~ 10%
	Calcium oxide	CaO	1305-78-8	<1 ~ 5%
	Iron oxide	Fe_2_O_3_	1309-37-1	<1 ~ 5%
	Ferrous oxide	FeO	1345-25-1	<1 ~ 5%
	Sodium oxide	Na_2_O	1313-59-3	<1 ~ 5%
Mixing solution	Magnesium chloride	MgCl_2_	7786-30-3	<1 ~ 50%
	Water	H_2_O		<1 ~ 50%

## Methods

### Preparation of MCM patch and tacrolimus ointment

The MCM patch used in this experiment was provided by MCM GLOBAL CO., Ltd. (Incheon, Korea). MCM patch was produced by pulverizing natural ore mineral containing tourmaline as the main materials. The stone flour was then mixed with magnesium chloride solution to induce aggregation reaction. Subsequently, the mixture was attached to a natural fabric which was then air- dried. The compositions of the ore powder are shown in Table 1. Tacrolimus ointment 0.1% (Protopic Co., Ltd. Osaka, Japan) was purchased at a pharmacy with hospital prescription and used as a positive control.

### Experimental setups and mouse categorization

Five-week-old female SKH-1 hairless mice with a mean weight of 25 ± 4.2 g were obtained from Orient Bio, Inc. (Seongnam, Republic of Korea) and kept in a suitable pathogen-free environment (22 ± 2 °C and 50 ± 10% humidity) under a 12-h light/dark cycle. Mice were provided free access to rodent chow food and filtered water until the end of the experiment. The mice were acclimatized for 1 week in spacious plastic cages (390 × 275 × 175 mm) with wood shaving bedding. Total sample size (*n* = 32, 4 groups) was calculated using the G*power program based on α error probability of 0.05 and power (1- β error probability) of 0.80, effect size 1, standard deviation (SD) 0.5. Thirty-two mice were randomly separated into four groups (*n* = 8 per group) as follows: normal control group without any treatment (NC), negative control treated with DNCB only (DNCB only), positive control group treated with DNCB and 0.1% tacrolimus ointment (PC), and experimental group treated with DNCB and MCM patch (MCM). Mice were identified by markers on the skin in their tail area. All experimental processes were performed in accordance with the protocol of the Institutional Animal Care and Use Committee, Yonsei University Wonju College of Medicine (ethical approval no: YWC-180615-2). To induce AD-like inflammation and skin lesions, we followed a previously described method with slight modifications [26]. Briefly, the dorsal skin (approximately 4 cm^2^) of all groups of mice except the NC group was treated with 200 μL of 1% DNCB (dissolved in a 3:1 mixture of acetone and olive oil) once a day for 1 week, and the dose was boosted by 150 μL of 0.5% DNCB every alternate day for 3 weeks. In the experimental group MCM patch was attached to the dorsal skin, fixed with medical tape (3 M™ elastic tape with liner; 3 M Science, USA) and changed every day for 7 days. In the PC group, tacrolimus ointment (0.1% Protopic Co. Ltd. Osaka, Japan) was applied to the AD-like lesional dorsal skin and taped once daily for 7 days. The DNCB only group were taped on the dorsal skin after induction of AD-like inflammation to generate the same stress level as that in the PC and MCM groups. At the end of the experiment, mice were anesthetized by using 2–3% isoflurane inhalation (Hana Pharm. Co., Hwaseong, Republic of Korea) at an oxygen flow rate of 0.5 L/min to minimize suffering and distress.

### Sample preparation

**Fig. 1:**
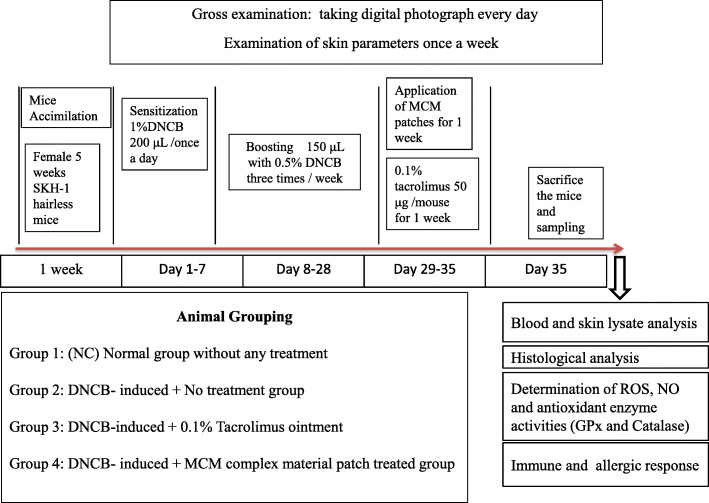
Schematic representation of the experimental procedure. AD-like inflammation was induced in the dorsal skin of female SKH-1 hairless mice with 1% DNCB for 1 week and 0.5% DNCB for 3 weeks. After 3 weeks of boosting, MCM patch and tacrolimus ointment were applied for 7 days to MCM and PC groups, respectively. On the 35th day, mice were sacrificed, and blood and skin tissue samples were collected for further analysis

All blood samples from mice were collected from the retro-orbital vein to EDTA coated BD microtainer tubes (Becton, Dickinson and Company, Franklin Lakes, NJ, USA) and kept in icepacks for 30 min. Immediately after blood collection, the mice were sacrificed by cervical dislocation. Further, serum was separated by centrifugation at 14, 000 rpm for 5 min at 4 °C. To obtain skin lysate, skin tissue was cut from the dorsal area of the mice and homogenized in ice-cold RIPA buffer (Pierce Biotechnology Inc., IL, USA) with protease inhibitor cocktail (Sigma Chemical Co., St Louis, USA) at 25 rpm for 15 min. Thereafter, the skin lysate was centrifuged at 14,000 rpm for 10 min at 4 °C and the obtained supernatant was checked for protein concentration using a Pierce™ BCA Protein Assay Kit (Thermo Scientific, Illinois, USA). Both collected serum and normalized skin lysate samples were stored at − 80 °C until use. A detailed timeline diagram for this experiment is shown in Fig. 1.

### Measurement of skin condition

The state of AD-like skin lesion including, scaling, erythema, erosion and edema, was macroscopically observed. Pictures were taken using a camera at a 1-week interval for 5 weeks. In addition, skin conditions of mice such as moisture level were measured by A-ONE TAB (Bomtech Electronics Co., L.td, Seoul, Republic of Korea). Besides that, skin barrier score, skin barrier strength, and TEWL were measured by Gp Skin (MSIP-CRM-G10 Barriersensor; Seoul, Republic of Korea). Briefly, the probe of GP Skin was placed at the center of the dorsal skin area for 5–10 s to record skin parameter results. For measuring barrier score and strength, two electronic sensors at the edge of the probe were placed to measure dielectric constant of stratum corneum of skin whereas pseudo-closed chamber (TEWL sensor) records TEWL. All measurement results were interpreted by GP skin device and directly sent to smart phone through Bluetooth for data accessibility. All skin conditions were non-invasively assessed by using skin diagnostic devices on the first, third and seventh day after MCM patch treatment. All measurements were performed in a very specific location on the dorsal area of the mice.

### Histological observation

The dorsal skin of hairless mice was collected for histological analysis. For light microscopical observation, the skin was cut, fixed in 10% neutral buffered formalin (0.1 M phosphate buffer, pH 7.4), dehydrated using a graded ethanol series, cleared by xylene and embedded in paraffin wax (Polyscience, Washington, USA). The paraffin section was cut into 4 μm- thick slices using a microtome (Reichert, Inc., New York, USA) and then stained with hematoxylin and eosin solution according to the routine method for measuring epidermal thickness. Epidermal thickness was measured in a randomly selected microscopic field of each slide at a regular interval for each mouse from each group. Measurements were performed using a microscopic software (Cell Sens Standard, Olympus, Tokyo, Japan) and microscopic observation was conducted under light a microscope (Olympus BX51, Olympus, Tokyo, Japan) at 100× magnification.

### Detection of intracellular total ROS level

To determine the effects of MCM on oxidative stress and superoxide level in serum and skin lysates, total ROS concentration was measured using ROS detection kit (Enzo Life Sciences Inc. New York, USA) according to a previously described method [21, 26, 36]. Briefly, serum, skin lysate (10 μL) and detection solution were mixed in a microplate well. The absorbance was measured using a DTX-800 multimode microplate reader (Beckman Counter, Inc., Fullerton, California, USA) using a filter set of 485/20 excitation and 528/20 emission.

### Detection of nitric oxide (NO) level

To determine the nitrite (NO_2_^−^) concentration in mouse serum and skin lysate, Griess reagent (Promega Corp., Madison, USA) was performed following a previously described method [21, 26, 36]. Briefly, 50 μL of serum was mixed with an equal volume of Griess reagent in a 96-well microplate and incubated at room temperature for 15 min. The absorbance was measured at 540 nm using the DTX-880 multimode microplate reader (Beckman Counter, Inc., Fullerton, California, USA). The NO_2_^−^ concentration was calculated by comparison with a standard curve graph generated by serial two-fold dilutions of sodium nitrate.

### Detection of endogenous antioxidant enzyme activities

To examine the antioxidant level in serum and skin lysate*,* the activity of GPx (Cayman’s GPx assay kit, Cayman Chemical Co., AnnArbor, MI USA) and CAT Biovision kit (Milpitas, California, USA) was measured by reading the absorbance at 340 nm using a the DTX-880 multimode microplate reader (Beckman Counter, Inc., Fullerton, California, USA) according to the manufacturer’s instruction.

### Immune profiling of serum inflammatory, Th_1_, and Th_2_ cytokines

The levels of inflammatory cytokines such as interleukin (IL)-1β, IL-6 and tumor necrosis factor (TNF)-α, in serum and skin lysate were measured. Moreover, the levels of Th_1_ cytokines including IL-2, IL-12 (p70), and interferon gamma (IFN)-γ, as well as Th_2_ cytokines including IL-4 and IL-10, were measured by using a multiplex array kit (Bio-Rad, San Diego, CA, USA) and run on Luminex technology (Bio-Plex Multiplex Bead Array System™, Bio-Rad, Hercules, CA, U.S.A.) according to manufacturer’s instruction. Raw fluorescence data were analyzed by using a five-parameter logistic method.

### Detection of total IgE level

Total IgE level in serum and skin lysate was measured using a sandwich enzyme-linked immunosorbent assay kit (BD Biosciences, San Diego, CA, USA) according to the manufacturer’s instructions. Reaction product was measured calorimetrically at 450 nm by a microplate reader (BioTek Instrument, Winooski, VT, USA).

### Statistical analysis

Data are shown as mean ± standard deviation (S.D.). Statistical analysis was performed by using analysis of variance (ANOVA) followed by multiple comparison test (Tukey’s post hoc test) using GraphPad Prism version 5.0 software packages (GraphPad Software, La Jolla, CA, USA). Differences were considered significant at *p* < 0.05.

## Results

### Effect of MCM treatment on the skin symptoms of AD-like skin lesion

**Fig. 2:**
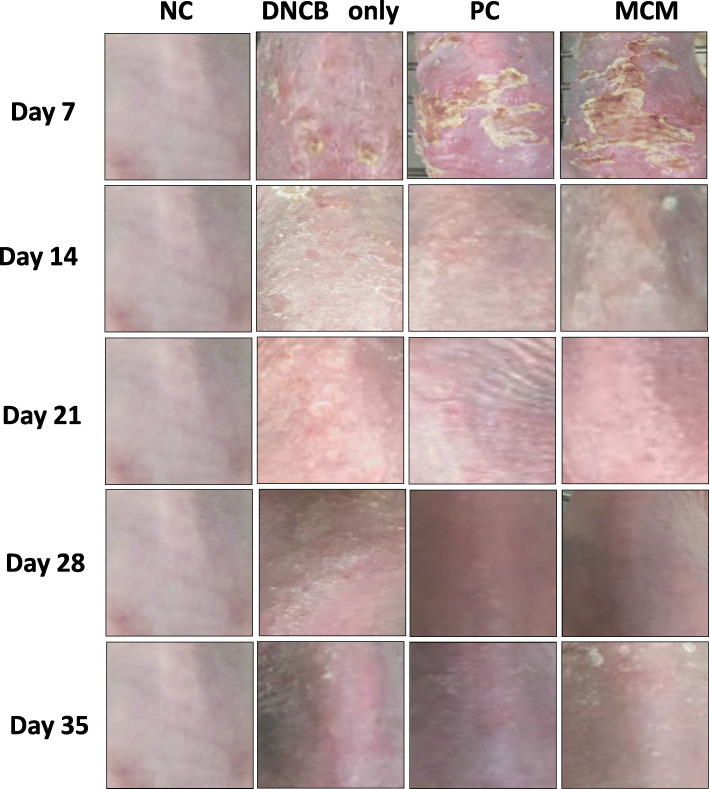
Macrographs of representing the skin condition of each group for 5 weeks. Representative skin images of each group captured every week for 5 weeks are shown. NC: normal control group without any treatment, DNCB only: negative control group treated with DNCB only, PC: positive control group treated with DNCB and tacrolimus ointment, and MCM: experimental group treated with DNCB and MCM patch

We examined whether MCM could inhibit DNCB-induced AD-like skin inflammation in SKH-1 hairless mice. In the macroscopical observation through representative dorsal skin lesion photographs for 5 weeks, we found that 4 weeks of DNCB treatment on the dorsal back skin induced AD-like skin lesions and symptoms including scaling, erythema, erosion and edema. However, skin symptoms in the MCM group were markedly improved after 7 days of topical application of MCM patch compared with those in the DNCB only group and the MCM group even showed better result than the PC group (Fig. 2).

### Skin test results after MCM treatment on AD-like skin lesions

To evaluate skin conditions after MCM patch treatment on the DNCB-induced AD-like inflammation and skin lesions, we tested four skin parameters. Our results revealed that skin barrier score was significantly higher on days 3 (*p* < 0.05) and 7 (*p* < 0.05) in the MCM group compared with that in the DNCB only group. In addition, our skin barrier strength results showed significant improvement in MCM group after days 1 (*p* < 0.05), 3 (*p* < 0.001) and 7 (*p* < 0.001) treatment compared with than in the DNCB only group. Likewise, the PC group also had significantly higher barrier strength score on days 3 (*p* < 0.05) and 7 (*p* < 0.05) of treatment compared with the DNCB only group. Skin moisture level was higher in the MCM group than in the PC and DNCB only groups, but the increase was not significant. In contrast, TEWL was significantly lower in MCM group than in the DNCB only group on days 3 (*p* < 0.001) and 7 (*p* < 0.001) of treatment. Likewise, the PC group also showed lower TEWL level on days 3 and 7 than in the DNCB only group but was not significant (Table 2).

**Table 2:** Skin conditions after MCM patch treatment during 7 days in DNCB-induced AD mice

Parameters	Groups	Treatment Days	3	7
		1		
**Barrier Score**	NC	80.1 ± 7.3 ^***^	69.4 ± 6.9 ^***^	74.6 ± 6.9 ^***^
	DNCB Only	63.7 ± 5.9	55.9 ± 5	53.8 ± 12.2
	PC	60.1 ± 4.5	57.3 ± 8.2	57.2 ± 7.8
	MCM	65.8 ± 2.3	64.7 ± 4.2 ^*^	63.5 ± 7 ^*^
**Barrier Strength**	NC	83.8 ± 2.9 ^***^	76 ± 4.1^***^	85.8 ± 3.4 ^***^
	DNCB Only	65.1 ± 9.5	53.6 ± 6.7	51.5 ± 22.9
	PC	63 ± 5.4	64.5 ± 6.7^*^	63.2 ± 7.8^*^
	MCM	76.9 ± 4.5 ^*^	75 ± 6.4^***^	72.8 ± 7.3 ^***^
**Moisture Level**	NC	77.1 ± 12.8 ^***^	61.6 ± 11.4^**^	59.6 ± 11.6
	DNCB Only	54.4 ± 5	50.2 ± 0.4	52.8 ± 5.7
	PC	50.2 ± 0.4	54.9 ± 10.3	50.3 ± 0.5
	MCM	50.3 ± 0.5	51.3 ± 3.2	55.7 ± 9
**Transepidermal Water Loss**	NC	7.3 ± 1.6^***^	10.5 ± 2.07^***^	6.4 ± 1.9^***^
	DNCB Only	15.9 ± 5.2	22.4 ± 3.3	22.2 ± 11.5
	PC	17.2 ± 3.2	19.6 ± 8.3	18.5 ± 3.4
	MCM	10.7 ± 2.1	11.1 ± 2.9^***^	12.8 ± 3.7^***^

*NC* Normal control group without any treatment, *DNCB only* Negative control group treated with DNCB only, *PC* Positive control group treated with DNCB and tacrolimus ointment, and *MCM* Experimental group treated with DNCB and MCM patch. Data are expressed as mean ± SD for 8 mice. Significance difference was analyzed with ANOVA and Tukey’s test. *p < 0.05, **p < 0.01, *** *p* < 0.001 vs. DNCB only group

### Skin histopathological findings after MCM patch treatment on DNCB-induced AD-like skin lesions

**Fig. 3:**
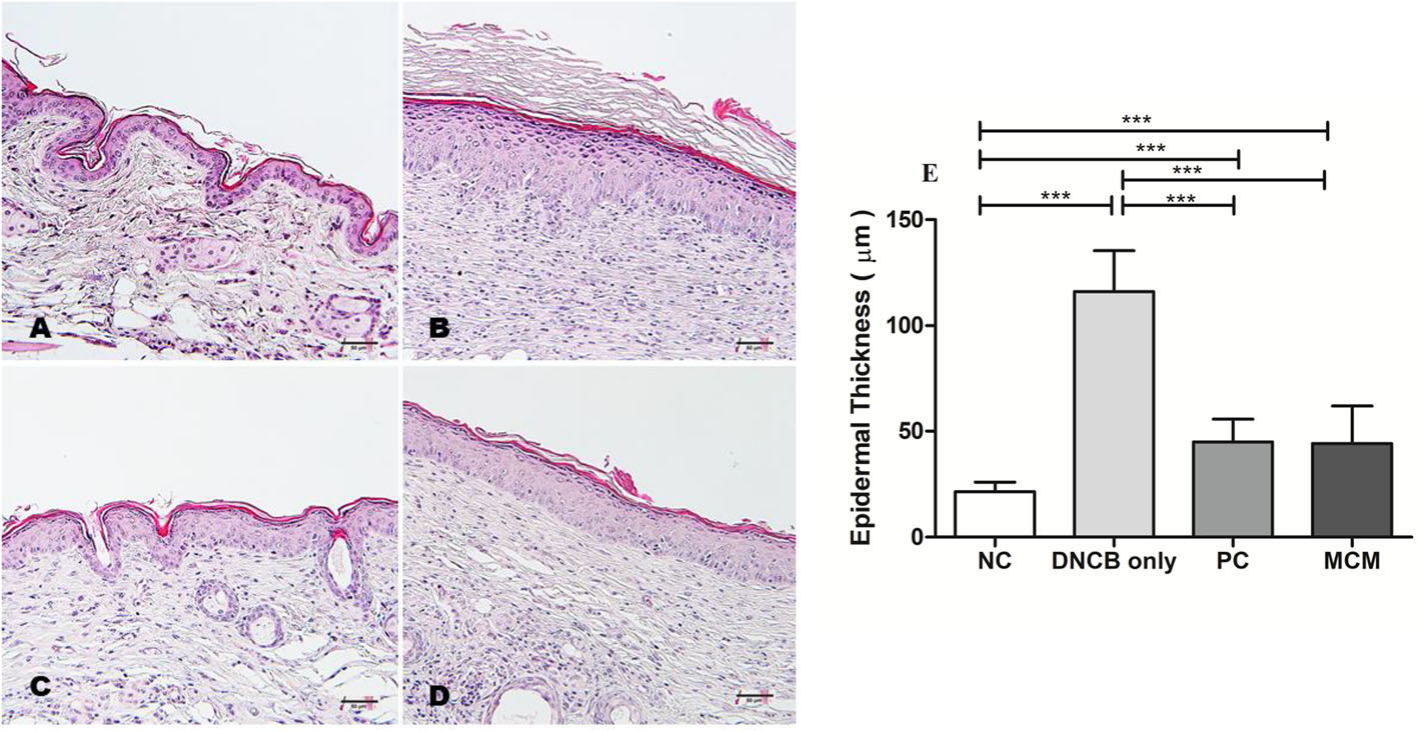
Histopathological features of the dorsal skin after MCM treatment in mice with DNCB-induced AD-like lesions. Representative macrographs of dorsal skin area and epidermal thickness are shown **a** Normal control group without any treatment. **b** Negative control group treated with DNCB only. **c** Positive control group treated with DNCB and tacrolimus ointment. **d** Experimental group treated with MCM patch after DNCB treatment. **e** Epidermal thickness. Hematoxylin-eosin staining, bar = 50 μm. Data are expressed as a mean ± SD. Significance difference was analyzed with ANOVA and Tukey’s test. *** *p* < 0.001

In the DNCB only group, the induced AD-like skin lesions showed the characteristics of epidermal hyperplasia including irregular line of the stratum basal layer and prominently increased epidermal thickness (116.1 ± 19.22 μm) compared with the NC group (21.55 ± 4.56 μm; Fig. 3a, b and e). However, MCM treatment markedly reduced epidermal thickness (44.22 ± 17.67 μm) by approximately 2.6 fold of that in the DNCB only group; the MCM group showed similar epidermal thickness as the PC group (44.93 ± 10.70 μm; Fig. 3c, d and e).

### Effects of MCM treatment on total intracellular ROS and NO levels in mice with DNCB-induced AD-like skin lesions

**Fig. 4:**
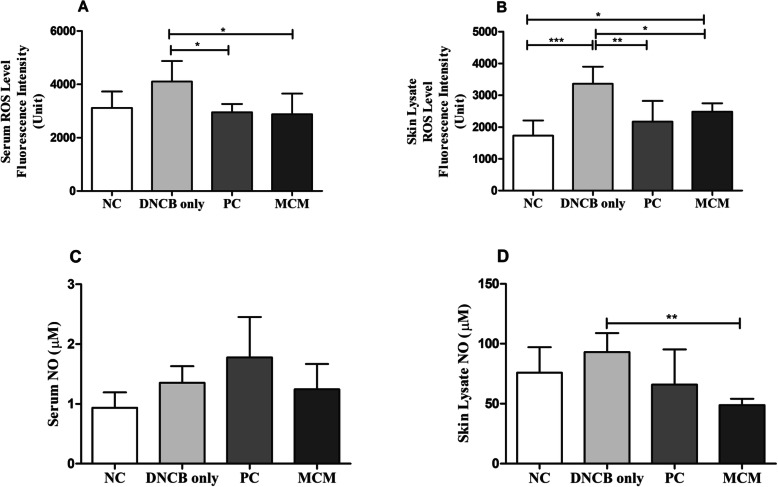
Total ROS and NO levels after MCM treatment in mice with DNCB-induced AD-like skin lesions and measured after 5 weeks. **a** Serum ROS. **b** Skin Lysate ROS. **c** Serum NO. **d** Skin Lysate NO levels. NC: normal control group without any treatment, DNCB only: negative control group treated with DNCB only, PC: positive control group treated with DNCB and tacrolimus ointment, and MCM: experimental group treated with DNCB and MCM patch. Data are expressed as a mean ± SD for 6 mice. Significance difference was analyzed with ANOVA and Tukey’s test. *p < 0.05, ** *p* < 0.01

To evaluate the effects of MCM treatment on DNCB-induced oxidative stress, we examined total intracellular ROS and NO levels in SKH-1 hairless mice. Our results showed that the total ROS levels in serum and skin lysate in both the PC and MCM groups were significantly decreased as compared with those in the DNCB only group (Fig. 4a and b). On the other hand, MCM skin lysate group showed significant decreased NO level (*p* < 0.01) than in the DNCB only group. In addition, we found that serum NO level was decreased in MCM group as compared to DNCB only group (Fig. 4c and d). These results indicates that application of MCM patch reduces the oxidative stress effectors such as ROS and NO and might alleviate the oxidative stress associated symptoms related with AD.

### Effects of MCM treatment on antioxidant enzymes (GPx and CAT) on serum and skin lysate

**Fig. 5:**
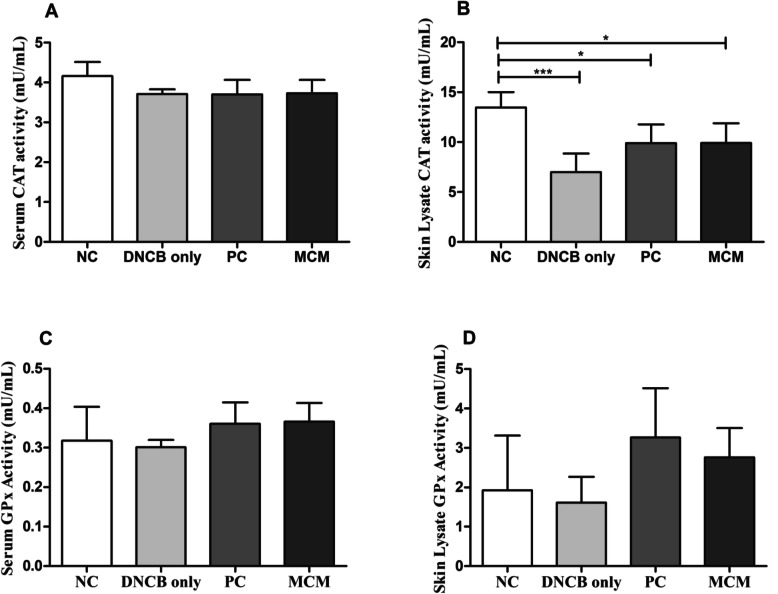
Changes in total anti-oxidant enzyme activities after MCM treatment in mice with DNCB-induced AD-like skin lesions. **a** Serum CAT. **b** Skin Lysate CAT. **c** Serum GPx. **d** Skin Lysate GPx. NC: normal control group without any treatment, DNCB only: negative control group treated with DNCB only, PC: positive control group treated with DNCB and tacrolimus ointment, and MCM: experimental group treated with DNCB and MCM patch. Data are expressed as mean ± SD for 6 mice. Significance difference was analyzed with ANOVA and Tukey’s test. *p < 0.05

We assessed the effect of MCM treatment on antioxidant enzyme activities in SKH-1 hairless mice with DNCB-induced AD-like skin lesions. Our results showed that CAT activity in skin lysate was significantly lower in the DNCB only group (*p* < 0.001), PC (*p* < 0.05), and MCM group (*p* < 0.05) than in the NC group, whereas serum CAT activity was not significantly different between all the groups (Fig. 5a and b). On the other hand, serum and skin lysate GPx activity in the MCM group and PC group were found higher as compared with DNCB only group (Fig. 5c and d). Our results showed that altered antioxidant enzyme activities were involved in the pathophysiology of exacerbation of AD. Of these, MCM application showed redox-balancing effects either systemic or regional against DNCB-induced AD-like skin lesions in mice.

### Effects of MCM patch on serum and skin lysate of cytokines

**Fig. 6:**
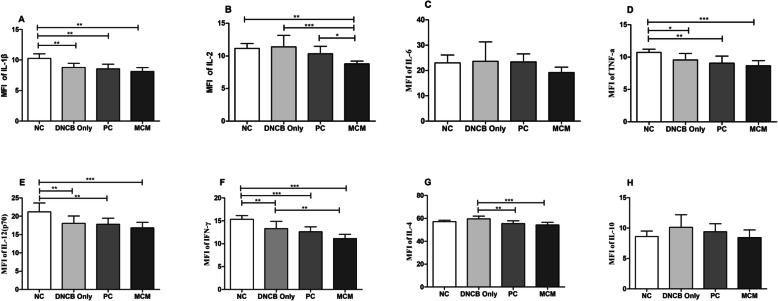
Inflammatory, Th_1_ and Th_2_ cytokines after MCM treatment in mice with DNCB-induced AD-like skin lesions in blood serum. The serum level of cytokines was measured with Bioplex Multiplex Bead array system. **a** IL-1β. **b** IL-2. **c** IL-6. **d** TNF-α. **e** IL-12 (p70). **f** IFN-γ. **g** IL-4. **h** IL-10. NC: normal control group without any treatment, DNCB only: negative control group treated with DNCB only, PC: positive control group treated with DNCB and tacrolimus ointment, and MCM: experimental group treated with DNCB and MCM patch. Data are expressed as mean ± SD for 8 mice. Significance difference was analyzed with ANOVA and Tukey’s test. *p < 0.05, **p < 0.01, ***p < 0.001

We investigated the relationship between topical application of MCM and AD-like skin lesions by examining various inflammatory, Th_1_, and Th_2_ cytokines closely associated with AD. In serum, we found that IL-1β level was markedly lower in DNCB only (*p <* 0.01), PC (*p <* 0.01) and MCM (*p <* 0.01) groups than in the NC group (Fig. 6a). Similarly, IL-2 level in the MCM group (*p <* 0.001) was significantly lower than in DNCB only group, PC group (*p <* 0.05) and NC (*p <* 0.01) group (Fig. 6b). Likewise, IL-6 level also showed a decreasing trend in the MCM group, but the decrease was not significant when compared with that in the control groups (Fig. 6c). In addition, we found that TNF-α was markedly lower in the MCM group (*p <* 0.001), PC group (*p <* 0.01) and DNCB only group (*p <* 0.05) than in NC group (Fig. 6d). Likewise, IL-12(p70) also showed significant lower level in the MCM group (*p <* 0.001), PC group (*p <* 0.01) and DNCB only group (*p <* 0.01) than in the NC group (Fig. 6e). IFN-γ level closely related with Th_1_ cell and was significantly reduced in MCM group (*p* < 0.01) than in the DNCB only group (Fig. 6f). However, we found IFN-γ was significantly lower in MCM group (*p <* 0.001), PC group (*p <* 0.001) and DNCB only group (*p <* 0.01) as compared to NC group (Fig. 6f). Next, we examined IL-4 activity which is related with Th_2_ cells, was significantly lower in the MCM (*p* < 0.001) and PC (*p* < 0.01) groups than in the DNCB only group (Fig. 6g). Lastly, we examined IL-10 activity and found slightly lower level in the MCM and PC groups but the reduction was not significant (Fig. 6h). Furthermore, in skin lysate, IL-4 level was significantly lower in the MCM group (*p <* 0.01) than in the DNCB only group (Supplementary Fig. 1 g). In contrast, IL-6 level was significantly higher in the MCM group (*p <* 0.01) than in the NC group (Supplementary Fig. 1c). However, the levels of other cytokines such as IL-1β, IL-2, TNF-α, IL-12 (p70), IFN-γ and IL-10 did not show significant differences (Supplementary Fig. 1). These results suggest that topical application of MCM modulate the Th_1_, and Th_2_ cytokines response and might alleviate the symptoms of AD- like skin lesion in mice.

### Effects of MCM treatment on IgE level in hairless mice on DNCB-induced AD-like skin lesions

**Fig. 7:**
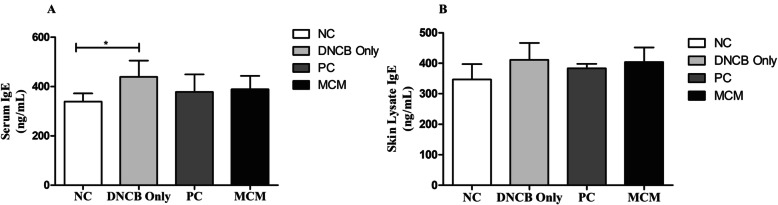
Total IgE levels after MCM treatment in mice with DNCB-induced AD-like skin lesions. IgE level of **a** serum and **b** skin lysate. NC: normal control group without any treatment, DNCB only: negative control group treated with DNCB only, PC: positive control group treated with DNCB and tacrolimus ointment, and MCM: experimental group treated with DNCB and MCM patch. Data are expressed as mean ± SD for 8 mice. Significance difference was analyzed with ANOVA and Tukey’s test. *p < 0.05

IgE is well-known as the most important indicators of AD. Therefore, we evaluated the total IgE concentration in the serum and skin lysate of SKH-1 hairless mice with DNCB-induced AD-like skin lesions. Our results revealed that the DNCB only group (*p* < 0.05) showed significantly higher activity of IgE level than the NC group in serum; however, the MCM and PC groups showed decrease in serum IgE level than the DNCB only group (Fig. 7a). Furthermore, IgE level in skin lysate did not show significant differences between all experimental groups (Fig. 7b).

## Discussion

This study showed that the therapeutic effects of MCM patch on DNCB-induced AD-lesions in hairless mice via regulation of skin barrier function and immune-redox balance. Concretely, this was evidenced further by improvement of morphological skin parameters such as TEWL and epidermal thickness, immune-redox balance, and suppression of allergic indicators. Of these, we first investigated skin morphology macroscopically. Recent studies have shown that disruption of skin barrier function contributes to the onset of AD and is the main cause of the subsequent release of pro-inflammatory mediators. Skin barrier disruption can increase skin susceptibility to various irritants and allergens, which in turn contributes to the pathogenesis of AD. To overcome this issue, researches have been ongoing to produce natural skin barrier protectors and emollients from natural products such as ore minerals that increase skin hydration to relieve the symptoms of atopic skin [18, 30, 31, 37–39]. In this study, we observed that repeated DNCB treatment of SKH-1 hairless mice skin decreased skin moisture level and skin barrier strength and increased TEWL level. In contrast, dermal administration of MCM patch led to a significant increase in skin barrier strength, and score as well as improved moisture level as shown in Table 2. Excessive dry skin and skin barrier dysfunction were associated with AD because low levels of ceramides accelerated TEWL and decreased water capacitance, ultimately resulting in atopic dry skin [40]. Our results revealed that topical application of MCM patch inhibited TEWL (Table 2), thereby improving skin condition. These results indicated that either natural or compounded complex ore minerals such as tourmaline might accelerate the recovery of skin barrier dysfunction by providing the vital micronutrients required for the regeneration of damaged skin tissues. Consistent with our results, several studies proven that tourmaline powder has beneficial physiological effects including acceleration of percutaneous blood circulation, sleep enhancement, and bacteriolysis [30–32].

Moreover, studies have reported that AD is characterized by epidermal hyperplasia, hyper production of keratin, intracellular edema and accumulation of lymphocytes and mast cells [38]. Therefore, we performed histological examination of the dorsal skin to measure epidermal thickness and to evaluate the extent of cell proliferation, hyperkeratosis, spongiosis, and epidermal hyperplasia. Our results revealed that distinctly increased epidermal thickness in AD-like skin lesions by DNCB-induction were decreased by MCM application and PC treatment as shown in Figs. 2 and 3. These findings suggested that MCM patch application improved the characteristic symptoms of AD. However, it is necessary to further clarify the effects of MCM patch on AD-like skin lesions through more in vivo and human clinical experiment.

Evidence has shown that allergic skin inflammation is mediated by oxidative stress and reduction in the levels of anti-oxidants [5, 26]. In particular, ROS are commonly known to be implicated in the development of many inflammatory diseases. As known, ROS are highly reactive and can interact with many biomolecules to destroy biological structures, thus promoting cellular damage and tissue destruction [41]. Macrophages are known to release proinflammatory mediators such as NO, which plays a vital role in aggravating inflammatory activities. However, RNS also plays a pivotal role in healing process from allergic inflammation and its regulation might be essential as it is known to affect the pathogenesis of several inflammatory diseases, including AD [42, 43]. To further verify whether MCM dermal application have effects on redox balance, analyses of ROS, NO, and antioxidants enzyme activities were conducted. Our results revealed that, ROS levels in both serum and skin lysate were elevated in the DNCB only group, whereas this level was found to be reduced in the MCM treated group both in serum and skin lysate (Fig. 4a and b). In addition, NO activity was found to be significantly increased with DNCB induction in DNCB only group. Interestingly, MCM patch treated group showed significantly decreased NO level in skin lysate as shown in Fig. 4d. Moreover, serum analysis showed decreased tendency in NO level (Fig. 4c). These significant reductions of oxidative stress markers such as ROS and NO might reveal the possible preventive effects of MCM treatment against tissue damages induced by DNCB in skin. Consistent with our ROS and NO results in DNCB only group, GPx activities in serum and skin lysate were found decreased. Moreover, we found higher level of GPx activity in MCM treated group in both serum and skin lysate (Fig. 5c and d). Higher GPx activity may play a protective role involved against oxidative stress via peroxidase scavenging [42–44]. Likewise, CAT activity in skin lysate was significantly lower in the DNCB only group (Fig. 5b). In contrast, CAT level were found increased tendency in NC, MCM and PC group in skin lysate but not in serum (Fig. 5). Therefore, increasing trend of antioxidant activities in skin lysate with MCM treatment might contribute to the reduction of oxidative effectors such as ROS and NO associated with AD.

To confirm this mechanism, we subsequently sought the immunological evidence of the effects of MCM by measuring serum IgE level in the SKH-1 hairless mice with DNCB-induced AD-like skin lesions. Elevated serum IgE level in serum is considered a hall mark of AD and as IgE promotes the clinical severity of AD by releasing inflammatory mediators [7, 8]. Consistent with our results on skin morphology, IgE level was increased in mice with DNCB only group, and MCM and PC treatments notably reduced serum IgE level (Fig. 7a). As IgE is a humoral reflection of Th_2_ immunity, our data suggest immunomodulation as a probable action mechanism of MCM. Various scientific mechanistic studies on AD have pointed to an imbalance between inflammatory, Th_1_ and Th_2_ responses. Interestingly, studies showed that AD is a biphasic inflammatory allergic skin disease that can predominantly secrete the Th_2_ cytokine like IL-4, which plays a pivotal role in acute phase in AD and responsible for B cell class switching to IgE production, whereas in the chronic phase in AD, Th_1_ cells secrete IL-2, IL-12, and IFN- γ, all of which plays a crucial role in the resolution of allergic-related immunopathology. Moreover, IL-2 participates in the pathogenesis of several pathological conditions including autoimmune and inflammatory diseases. Therefore, Th_1_ and Th_2_ imbalance plays an important role in AD development [45–50]. Repeated DNCB sensitization promotes the release of both Th_1_ and Th_2_ cytokines by activating helper T cells [39]. A previous study reported that IFN- γ expression in mice with chronic AD-like skin lesions was elevated to similar levels as that in humans with AD lesions [47]. Furthermore, in our study, dermal application of MCM patch suppressed the systemic levels of IL-4, IL-2, and IFN- γ in AD-like skin lesions (Fig. 7), suggesting that MCM controls inflammatory and allergic responses via Th_1_/Th_2_ regulation of cytokines. Likewise, in skin lysate the production of IL-4 was reduced with MCM patch treatment could be considered as a possible mechanism of anti-apoptotic effect of MCM application (Supplementary Fig. 1).

Pro-inflammatory cytokines such as TNF-α and IL-1β are well-known pathogenic inducers of AD. Moreover, IL-6 and IL-10 act as both pro-inflammatory and anti-inflammatory cytokines, which are promptly produced in various inflammatory diseases. Several researchers have observed increased levels of IL-6 and IL-10 in AD lesional skin [41–43]. In the present study systemic cytokines level such as TNF-α, IL-1β, IL-6, and IL-12 expression were found decreased in DNCB-induced AD-like skin lesions (Fig. 6). In addition, MCM application showed slight reduction of these pro-inflammatory cytokines. These results indicated that MCM patch might alleviate the pathogenesis of AD through regulation of various inflammatory cytokines. However, further in-depth studies are required to fully clarified this notion.

## Conclusion

Collectively, our results showed that MCM treatment suppressed AD-like skin lesions by increasing skin barrier score and strength and skin moisture level, as well as, by decreasing the TEWL. Moreover, MCM patch dermal application also reduced the levels of the oxidative stress markers such as ROS and NO level in skin lysate and showed increasing tendency of the total anti-oxidant enzyme activities. In addition, histological results showed that MCM application significantly decreased DNCB-induced epidermal thickness in AD-like skin lesions. Furthermore, MCM application also inhibited Th_1_/Th_2_ and inflammatory cytokines. Taken together, our findings indicated that MCM patch treatment might have potential effective regional as well as systemic effects against AD lesional skin by regulating the skin barrier function and balancing immune-redox responses. However, further studies should investigate how MCM works at the molecular level by targeting signaling pathways and inflammatory cell production because AD is an oxidative stress-related inflammatory disease.

## Supplementary Information

**Additional file 1: Supplementary Fig. 1.** Inflammatory, Th_1_ and Th_2_ cytokines after MCM treatment in mice with DNCB-induced AD-like skin lesions in skin lysate. Cytokines levels of skin lysate were measured with Bioplex Multiplex Bead array system. **a** IL-1β. **b** IL-2. **c** IL-6. **d** TNF-α. **e** IL-12 (p70). **f** IFN-γ. **g** IL-4. **h** IL-10. NC: normal control group without any treatment, DNCB only: negative control group treated with DNCB only, PC: positive control group treated with DNCB and tacrolimus ointment, and MCM: experimental group treated with DNCB and MCM patch. Data are expressed as mean ± SD for 8 mice. Significance difference was analyzed with ANOVA and Tukey’s test. **p < 0.01.

## Data Availability

The datasets supporting the conclusions of this article are included within the article.

## Abbreviations

AD: Atopic dermatitis
ANOVA: Analysis of variance
CAT: Catalase
DNCB: 2, 4-dinitrochlorobenzene
DNCB only: Negative control treated with DNCB only
GPx: Glutathione peroxidase
IgE: Immunoglobulin E
IFN γ: Interferon γ
IL: Interleukin
MCM: Mineral complex material
NC: Normal control group without any treatment
PC: 0.1% tacrolimus ointment
ROS: Reactive oxygen species
RNS: Reactive nitrogen species
TEWL: Trans-epidermal water loss
Th1: T helper cell type 1
Th2: T helper cell type 2
TNF: Tumor necrosis factor
