# Supplementary figures and images for: Effects of mineral complex material treatment on 2,4- dinitrochlorobenzene-induced atopic dermatitis like-skin lesions in mice model
Source: BMC Complement Med Ther. 2021 Mar 3;21:82. doi: 10.1186/s12906-021-03259-5 (PMC7931355; doi:10.1186/s12906-021-03259-5)

**Supplementary Fig.1**


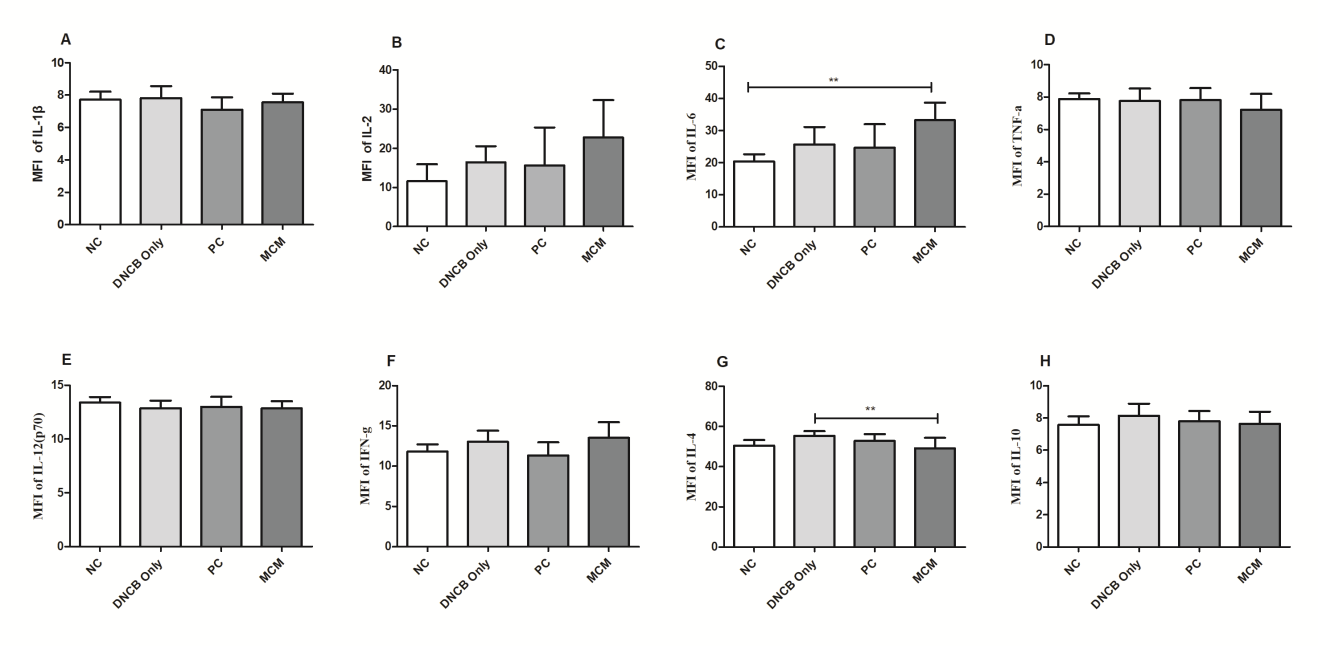

Supplement: Supplementary file 1 — Additional file 1: Supplementary Fig. 1. Inflammatory, Th1 and Th2 cytokines after MCM treatment in mice with DNCB-induced AD-like skin lesions in skin lysate. Cytokines levels of skin lysate were measured with Bioplex Multiplex Bead array system. a IL-1β. b IL-2. c IL-6. d TNF-α. e IL-12 (p70). f IFN-γ. g IL-4. h IL-10. NC: normal control group without any treatment, DNCB only: negative control group treated with DNCB only, PC: positive control group treated with DNCB and tacrolimus ointment, and MCM: experimental group treated with DNCB and MCM patch. Data are expressed as mean ± SD for 8 mice. Significance difference was analyzed with ANOVA and Tukey’s test. **p < 0.01. [file 12906_2021_3259_MOESM1_ESM.docx]
